# Genome Sequence Analysis of Native *Xenorhabdus* Strains Isolated from Entomopathogenic Nematodes in Argentina

**DOI:** 10.3390/toxins16020108

**Published:** 2024-02-17

**Authors:** Leopoldo Palma, Laureano Frizzo, Sebastian Kaiser, Colin Berry, Primitivo Caballero, Helge B. Bode, Eleodoro Eduardo Del Valle

**Affiliations:** 1 Instituto de Biotecnología y Biomedicina (BIOTECMED), Departamento de Genética, Universitat de València, 46100 Burjassot, Spain; 2 Consejo Nacional de Investigaciones Científicas y Técnicas (CONICET), Ciudad Autónoma de Buenos Aires C1033AAJ, Argentina; 3 Instituto Multidisciplinario de Investigación y Transferencia Agroalimentaria y Biotecnológica (IMITAB), Consejo Nacional de Investigaciones Científicas y Técnicas (CONICET), Universidad Nacional de Villa María (UNVM), Villa María 1555, Argentina; 4 ICIVET Litoral, CONICET-UNL, Departamento de Salud Pública, Facultad de Ciencias Veterinarias, Universidad Nacional del Litoral, Esperanza S3080, Argentina; lfrizzo@fcv.unl.edu.ar; 5 Department of Natural Products in Organismic Interactions, Max-Planck-Institute for Terrestrial Microbiology, 35043 Marburg, Germany; sebastian.kaiser@mpi-marburg.mpg.de (S.K.); Helge.Bode@mpi-marburg.mpg.de (H.B.B.); 6 Evolutionary Biochemistry Group, Max-Planck-Institute for Terrestrial Microbiology, 35043 Marburg, Germany; 7 Cardiff School of Biosciences, Cardiff University, Museum Avenue, Cardiff CF10 3AX, UK; berry@cardiff.ac.uk; 8 Institute for Multidisciplinary Research in Applied Biology, Universidad Pública de Navarra, 31006 Pamplona, Spain; pcm92@unavarra.es; 9 Departamento de Investigación y Desarrollo, Bioinsectis SL, Polígono Industrial Mocholi Plaza Cein 5, Nave A14, 31110 Noain, Spain; 10 Molecular Biotechnology, Department of Biosciences, Goethe Universität Frankfurt, 60438 Frankfurt, Germany; 11 Center for Synthetic Microbiology (SYNMIKRO), Phillips University Marburg, 35043 Marburg, Germany; 12 Department of Chemistry, Phillips University Marburg, 35043 Marburg, Germany; 13 Senckenberg Gesellschaft für Naturforschung, 60325 Frankfurt, Germany; 14 ICiagro Litoral, CONICET, Facultad de Ciencias Agrarias, Universidad Nacional del Litoral, Kreder 2805, Esperanza S3080, Argentina

**Keywords:** *Xenorhabdus*, entomopathogenic nematodes, genomic sequence, virulence factors, insecticidal proteins, secondary metabolites, phylogenetics, bioinsecticides, insect pests, biocontrol

## Abstract

Entomopathogenic nematodes from the genus *Steinernema* (Nematoda: Steinernematidae) are capable of causing the rapid killing of insect hosts, facilitated by their association with symbiotic Gram-negative bacteria in the genus *Xenorhabdus* (Enterobacterales: Morganellaceae), positioning them as interesting candidate tools for the control of insect pests. In spite of this, only a limited number of species from this bacterial genus have been identified from their nematode hosts and their insecticidal properties documented. This study aimed to perform the genome sequence analysis of fourteen *Xenorhabdus* strains that were isolated from *Steinernema* nematodes in Argentina. All of the strains were found to be able of killing 7th instar larvae of *Galleria mellonella* (L.) (Lepidoptera: Pyralidae). Their sequenced genomes harbour 110 putative insecticidal proteins including Tc, Txp, Mcf, Pra/Prb and App homologs, plus other virulence factors such as putative nematocidal proteins, chitinases and secondary metabolite gene clusters for the synthesis of different bioactive compounds. Maximum-likelihood phylogenetic analysis plus average nucleotide identity calculations strongly suggested that three strains should be considered novel species. The species name for strains PSL and Reich (same species according to % ANI) is proposed as *Xenorhabdus littoralis* sp. nov., whereas strain 12 is proposed as *Xenorhabdus santafensis* sp. nov. In this work, we present a dual insight into the biocidal potential and diversity of the *Xenorhabdus* genus, demonstrated by different numbers of putative insecticidal genes and biosynthetic gene clusters, along with a fresh exploration of the species within this genus.

## 1. Introduction

The (entomopathogenic) nematodes from the *Steinernema* (Nematoda: Steinernematidae) genus have the ability to infest and result in the rapid killing of insect hosts in mutualistic association with their Gram-negative symbiont bacteria belonging to the genus *Xenorhabdus* (Enterobacterales: Morganellaceae) [1,2]. The nematodes track and infest soil-dwelling insect larvae, penetrating through natural openings such as the anus, the mouth, or the spiracles. Once the nematodes reach the haemocoel, the bacteria are released by regurgitation into the insect blood (haemolymph). At this moment, the bacteria deliver different virulence factors that kill the insect host rapidly, by means of massive toxaemia and septicaemia [3]. These virulence factors encompass different insecticidal proteins (e.g., Tc, Txp, Mcf, Pra/Prb and App), enzymes (e.g., chitinases and proteases) and secondary metabolites produced at the stationary growth phase, which kill the insect while also inhibiting other opportunistic microorganisms [4]. Following the establishment of a nutritive and safe environment, the entomopathogenic nematode reproduces, and newly formed infective juveniles (IJs) acquire the symbiotic bacteria after feeding, which is followed by abandonment of the depleted insect body to seek new prey [5,6].

These characteristics have positioned entomopathogenic nematodes as valuable assets in the field of the biological control of insect pests and, consequently, they have been mass-produced on a large scale and are commercially available [7,8]. 

To date, the NCBI Taxonomy Database [9] assigns *Xenorhabdus* strains into only 30 defined species; however, another 162 additional strains, including those described in this study, remain still unassigned to a species and are described as unclassified *Xenorhabdus* species (https://www.ncbi.nlm.nih.gov/Taxonomy/Browser/wwwtax.cgi?id=626, accessed on 31 January 2024). 

A diverse range of genes encoding putative insecticidal proteins produced by *Xenorhabdus* and *Photorhabdus* (Enterobacterales: Morganellaceae) species (e.g., Tc, Txp, Mcf, Pra/Prb and App proteins) are attracting attention in the scientific community, since some may be promising biological control tools for the construction of innovative next-generation insect-resistant crops [10,11]. Toxin complex (Tc) homologs may be found in *Photorhabdus*, *Xenorhabdus* and *Yersinia entomophaga* (Enterobacterales: Yersiniaceae) [12], and are formed by three subunits called A, B and C (e.g., TcA, TcB and TcC). Subunit A is involved in the binding of the toxin receptor and also participates in the translocation of the toxin, the B subunit participates as the linker among subunits, and the C subunit solely exhibits specific toxic activity (ADP-ribosyltransferase in some cases, although there is a diversity of C subunit activities). These insecticidal proteins have been shown to be active against a wide range of insects [13], including the lepidopteran *Galleria mellonella* (L.) (Lepidoptera: Pyralidae). Mcf (makes caterpillars floppy) toxin provokes the apoptosis of haemocytes and has shown activity against *Manduca sexta* (L.) (Lepidoptera: Sphingidae) [14]. Pra/Prb (formerly PirA/PirB) exhibit some similarity with Cry toxins from *Bacillus thuringiensis* (Berliner) (Bacillales: Bacillaceae) and have shown activity against *G. mellonella* by injection and against *Plutella xylostella* (L.) (Lepidoptera: Plutellidae) by ingestion [15]. Lastly, App1Ba1/App2Ba1 toxins (previously called XaxA/XaxB) are two-component α-pore-forming toxins where App1Ba1 activates and stabilizes App2Ba1 before the two form a heteromer of 13 subunits each, in which the App2 protein contributes to the transmembrane regions of the pore [16]. Other virulence factors produced by *Xenorhabdus* species include a novel insecticidal protein class named Txp showing toxic activity by injection against the lepidopterans *G. mellonella*, *Helicoverpa armigera* (Persoon) (Lepidoptera: Noctuidae) and *Plodia interpunctella* (Hübner) (Lepidoptera: Pyralidae) [17], nematocidal proteins and chitinase enzymes [10], plus chitin-binding proteins [18], which may contribute not only to killing the insect host but also to inhibiting other nematode and fungal competitors that may jeopardize the normal development of the entomopathogenic nematode.

In addition, some species of *Xenorhabdus* have been reported to synergize the insecticidal activity of *B. thuringiensis* against some insect pests such as mosquitoes [19,20,21], turning them into potential supplementary ingredients for improving *B. thuringiensis*-based insecticides.

As mentioned above, *Xenorhabdus* species are also capable of producing secondary metabolites with biological activity for the benefit of both the nematode host and the symbiotic bacteria, since they display antimicrobial (antibacterial and antifungal) and anti-parasitic activities [22]. These metabolites are synthesised by the bacterium between the late exponential growth phase and the beginning of the stationary growth phase [23], with their synthetic pathways commonly encoded at different gene clusters [22]. 

The aim of this work was to perform the genome sequence analysis of fourteen new *Xenorhabdus* strains isolated in Argentina in order to provide an updated insight into the *Xenorhabdus* genus and its diversity, as well as report their potential for the production of insecticidal proteins and bioactive compounds. 

## 2. Results

### 2.1. Isolation of Bacteria and Preliminary Identification

All nematode hosts were able to kill 7th instar *G. mellonella* larvae at a maximum of 48 *h* after exposure. The axenic isolation of bacteria rendered fourteen isolates exhibiting typical colours on NBTA agar plates, which were preliminarily classified as *Xenorhabdus* species, as per their high % pairwise similarity of amplified 16S rRNA sequences (Table 1). The isolation of strains 42, M, Cul and 38 was from nematodes taxonomically classified as the species *Steinernema rarum* (Poinar, Jackson & Klein) (Rhabditida: Steinernematidae), whereas the isolation of strains 18 and DI was from nematodes classified as *Steinernema diaprepesi* (Nguyen and Duncan) (Rhabditida:Steinernematidae). The isolation of the remaining strains, namely Flor, 5, PSL, Reich, Vera, 12, 3 and ZM, was from *Steneinerma* sp. nematodes that were not classified to species level (Table 1). 

### 2.2. Genome Assembly and Phylogenetic Analysis

A summary of key attributes from the assembled genomic sequences is presented in Table 3. The genome sizes ranged from 4,070,051 to 4,937,636 base pairs (bp), with a % G+C content ranging from 42.8 to 45.4, showing consistency with both the genome size and % G+C from other documented *Xenorhabdus* strains [24,25,26,27]. The RAST server estimation of the number of predicted coding sequences (CDSs) ranged between 3748 and 4499.

**Figure 1:**
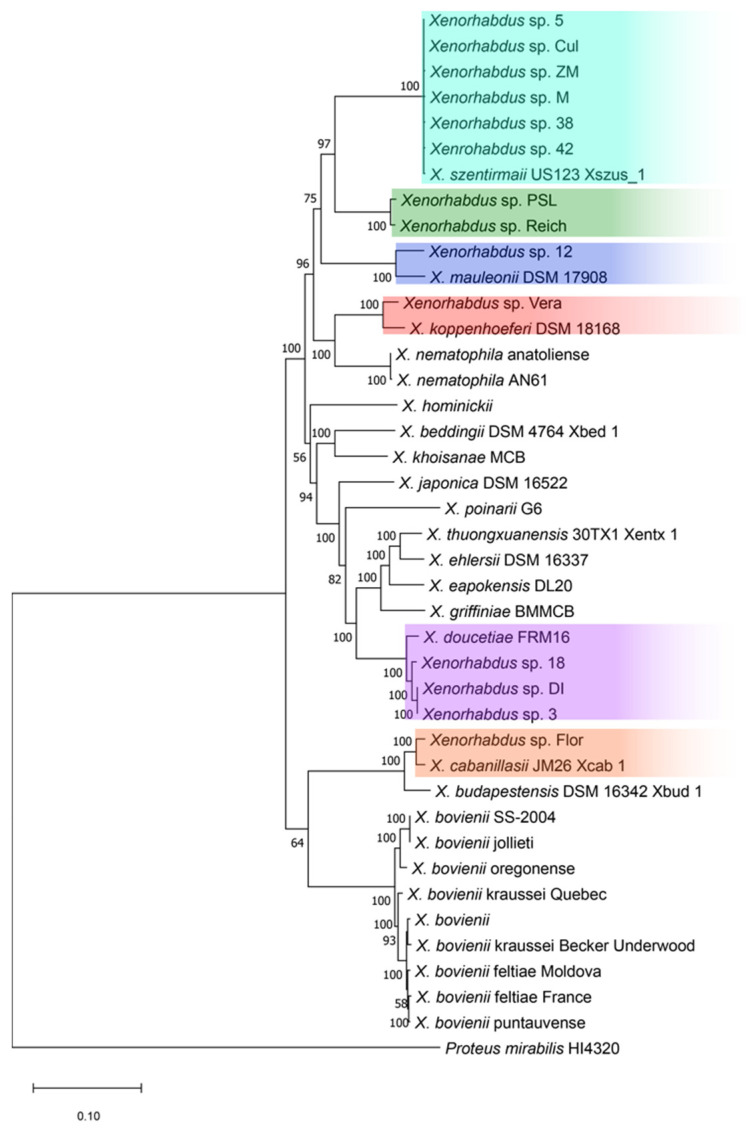
Maximum-likelihood tree (GTR+G+I) for the 41-taxon dataset. Likelihood bootstrap values were determined from 100 replicates and are indicated at each branch. *Proteus mirabilis* (Enterobacterales: Morganellaceae) strain HI4320 was chosen as out-group. Different colours highlight species clustered in different groups.

A maximum-likelihood phylogenetic tree was constructed using concatenated housekeeping genes. The tree showed that strains 5, Cul, ZM, M, 38 and 42 form, together with *X. szentirmaii* US123 Xszus_1 (Acc. no. NIUA01000001.1), a monophyletic group, suggesting then that they belong to the same species (Figure 1). This finding was consistent with the high ANI values (>95%) shown when the *X. szentirmaii* US123 Xszus_1 genome was used as the reference sequence, and was confirmed using the TYGS server (Table 2). The multiple-gene phylogenetic approach also grouped strains PSL and Reich separately from the rest of the species, suggesting that they should represent a novel *Xenorhabdus* species (Figure 1). This result was consistent with a calculated % ANI below 95% and was confirmed by the TYGS server, which failed to identify species matches for the PSL and Reich strains (Table 2). *Xenorhabdus* strain 12 was most closely related to *X. mauleonii* DSM 17908; however, the calculated %ANI was 90.23 between these two isolates and below 85.00% ANI with the rest of the strains, suggesting that strain 12 should correspond to a novel species. This finding was confirmed by the TYGS server, which also failed to identify strain 12. Strain Vera was grouped with *X. koppenhoeferi* DSM 18,168 and also exhibited a consistent ANI value >95%, plus the corresponding reliable identification using the TYGS server, which also identified the species as *X. koppenhoeferi*. Strains 18, DI and 3 were clustered together with *X. doucetiae* strain FRM16, showing consistent ANI values and a TYGS analysis supporting species identification. Lastly, strain Flor was grouped with *X. cabanillasii* JM26 Xcab_1 with an ANI value >97%, and the same result was provided by the TYGS server.

### 2.3. Gene Prediction and Annotation of Virulence Factors

A total of 110 putative insecticidal genes were identified in the genomes. These genes showed a diverse degree of pairwise similarity with known insecticidal proteins from other Gram-negative entomopathogenic bacteria, including *Photorhabdus luminescens*, *X. nematophila*, and *Yersina entomophaga* (Table 3). Furthermore, our strains also contained additional virulence factors, including potential chitinases and nematocidal proteins (nProteins) derived from *Xenorhabdus bovienii*. Insecticidal protein counterparts exhibited a notable resemblance to toxin complex (Tc) subunits, such as Xpta1 proteins from *X. nematophila* [28] and Yen-toxin complex proteins from *Y. entomophaga* [29]. In addition, Pra/Prb homolog proteins, previously known as PirA/PirB (*Photorhabdus* insect-related proteins) before the BPPRC nomenclature revision [30], and Mcf1 (makes caterpillars floppy) homolog proteins from *P. luminescens* were also found [31]. A few less representative insecticidal proteins were found to have significant similarity with App1B proteins (formerly XaxA proteins) [30,32]. We also found the more recently described Txp class homolog proteins encoded in the genomes of strains PSL, Vera and Flor (Table 3). Within our dataset, genes encoding Txp proteins appear absent from *X. szentirmaii, X. doucetiae* and *X. santafensis* strains; Pra/Prb are absent from *X. szentirmaii, X. littoralis* and *X. santafensis* strains; and App is absent from *X. littoralis*, *X. koppenhoeferi* and *X. doucetiae* strains. 

**Figure 2:**
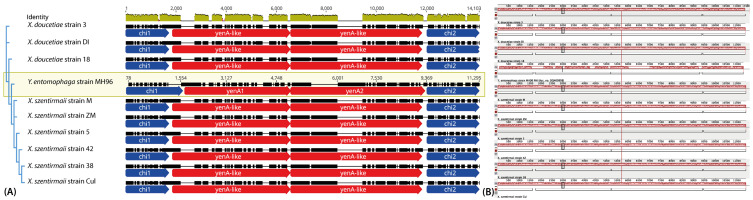
Comparison of pathogenicity island regions which show collinearity with the *Y. entomophaga* MH96 PAI_Ye96_ (Acc. no. DQ400808) strain used as the reference sequence [29]. The *yen*-like gene sequences encode toxin complex homolog proteins. (**A**) Schematic representation of MAUVE multiple sequence alignments showing sequence collinearity among pathogenicity islands including flanking predicted chitinase and inner *yen*-like genes (Tc homologs) among *Y. entomophaga* strain MH96 and *Xenorhabdus* strains. The tree on the left shows that this region is conserved with regard to species classification and that the *Y. entomophaga* MH96 sequence is closely related. (**B**) MAUVE representation of the collinear block. Matches are shared in a single non-partitioned block of identical colour which indicates the full collinearity of the matching region. A unique connecting line is consistent with the alignment of the putative PAIs into one colinear block.

**Figure 3:**
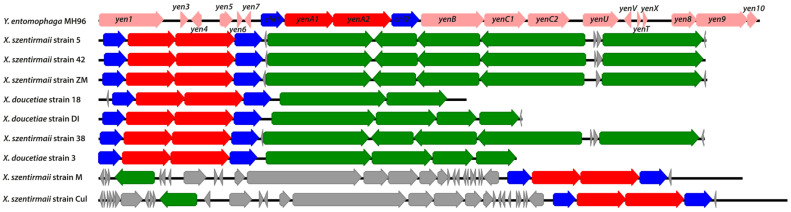
Schematic representation of the pathogenicity island from *Y. entomophaga* MH96 and the homologous regions found in putative pathogenicity islands from *Xenorhabdus* genomes. Note: The pathogenicity islands related to the *chi1*, *yenA1*, *yenA2* and *chi2* coding regions are not aligned so that other flanking regions can be illustrated. Genes encoding *chi* and *yen* homologs are coloured blue and red, respectively (the common *chi1*-*yenA1*-*yenA2*-*chi2* motif from MH96 strain is coloured in dark blue/red to highlight the region of homology); other insecticidal genes in *Xenorhabdus* are coloured green and other non-related genes are coloured grey.

Strains 3, DI and 18 corresponding to *X. doucetiae* species and strains M, ZM, 5, 42, 38 and Cul corresponding to *X. szentirmaii* species showed an ~11 kb region encoding two putative chitinases flanking two yen-like homolog genes. Multiple sequence alignments performed using MAUVE [33] rendered a single colinear block conserved among all of these *Xenorhabdus* genome regions, and also a region from the genome of *Y. entomophaga* MH96 (Figure 2). In *Y. entomophaga* MH96, this feature is part of an ~47 kb pathogenicity island [29], and it is interesting to note that in the nine *Xenorhabdus* genomes above, there is also a clustering of putative virulence genes around this feature (Figure 3). On the other hand, strains Flor, PSL, Vera, Reich and 12 exhibited their predicted insecticidal genes, along with other encoded virulence factors, distributed across the genome sequences.

### 2.4. Prediction of Biosynthetic Gene Clusters

The biosynthetic gene cluster analyses using antiSMASH [34] showed a wide diversity of gene clusters that could potentially be involved in the production of several bioactive secondary metabolites in the *Xenorhabdus* strains (Table 4). The gene clusters used as reference have been experimentally reported in the scientific literature. Their functions are described in Table 4 and include the production of siderophores (photobactin and photoxenobactin), antioxidative pigments (aryl polyenes), antimicrobial products (xenobactin and fabclavine), antibiotics (phenazine, pyrrolizixenamide, xenocoumacin and safracin), possible insecticidal roles (gameXPeptide, lipocitide, photoxenobactin, xefoampeptide and xenotetrapeptide), putrebactin/avaroferrin and antifungal compounds (PAX lipopeptides, xenocoumacin). Other functions include inhibiting proteasomes and involvement in quorum sensing and iron uptake, in addition to others currently of unknown function (rhabdobranin, szentirazine and ATRed). 

The strains showed unique profiles regarding the number and variety of the encoded gene clusters, exhibiting both intra and interspecific differences. Strain DI showed the lowest number of gene clusters with eight clusters (including a duplication for the synthesis of putrebactin/avaroferrin), along with strain 3 with nine gene clusters and also a duplicated putrebactin/avaroferrin cluster. In contrast to the report of Tobias et al. (2017) on several *Xenorhabdus* strains, the new strains analysed here do not encode GxpS for the synthesis of gameXPeptide, and do not produce either XfpS (xefoampeptides) or XtpS (xenotetrapeptide), with the exception of the Vera strain, which is unique in showing the presence of the Xtp cluster and, therefore, may be capable of xenotetrapeptide synthesis. The genome of strain Cul showed the greatest diversity of predicted gene clusters with eighteen gene clusters in total. Strains Cul, 38 and 42 (that belong to the same species) stand out from the rest, since they exhibit two cluster sequences for phenazine and ATRed clusters, whereas strain Reich is distinguished because it is the only one showing three duplications for the ATRed cluster. In strains Cul, M, 38, 42, PSL, 12 and Flor, the ATRed cluster was found more than once in their genomic sequences (Table 4).

## 3. Discussion

Entomopathogenic bacteria beyond *B. thuringiensis* are rapidly emerging as novel and promising resources for sustainable pest control in modern agriculture. For instance, Gram-negative bacteria including *Y. entomophaga*, *Yersinia pestis* and *Pseudomonas entomophila* are currently gaining attention because of their interesting insecticidal activity against insects of different orders [36]. One recent example is the Gram-negative bacterium *Chromobacterium piscinae* (Neisseriales: Neisseriaceae), which demonstrated its capability for producing insecticidal proteins able to kill larvae of the Western corn rootworm *Diabrotica virgifera virgifera* (LeConte) (Coleoptera: Chrysomelidae), a severe maize-specific pest causing significant crop losses in North America and Europe [37,38].

The bacterial strains reported in this work were able, along with their nematode counterparts, to kill *G. mellonella* larvae (7th instar) in just two days of exposure. Although we have not tested the toxicity of any bacterial insecticidal protein from the bacterial strains yet, the mortality exhibited by *G. mellonella* larvae strongly suggests insecticidal activity from the symbiotic bacteria. *X. nematophila* has been proven to be extremely pathogenic for *G. mellonella* larvae after inoculation, even at very low concentrations [12], and other strains of this genus have also been reported to be lethal to insects by injection in the absence of the symbiotic nematode [12], in some cases being more efficient than the nematode itself for killing insects. Despite the fact that *Steneinerma* nematodes devoid of their symbiotic bacterium are still able to kill their hosts [39], only nematode–bacterial complexes can produce the fast killing of the insect within two days, demonstrating the remarkable entomopathogenic role of their bacterial counterpart [40]. 

Prb/Pra binary proteins are toxic by ingestion and may have utility as biological assets for the control of lepidopteran pests including *P. xylostella* [41], a species with evolved resistance to both Bt-formulated insecticides and some Cry proteins used in Bt crops (e.g., Cry1Ac) [42]. These binary proteins are also active against human disease vectors including *Aedes aegypti* and *Aedes albopictus* (L.) (Diptera: Culicidae), causing no harm to *Mesocyclops thermocyclopoides* (Cyclopoida: Cyclopidae), which is a predator of mosquitoes [43]. 

In contrast, Tc toxin complexes App (formerly Pax, Xax and Yax) and Mcf (makes caterpillars floppy) are not likely to be used as biological control agents. Tc toxins are orally active, heterotrimeric protein complexes that require A, B and C components for full toxicity. The fact that Tc toxins are large heterotrimeric insecticidal proteins with high molecular weight may render their expression in plants inefficient and the use of enzyme-mediated toxicity is not favoured. Mcf [14] toxins have been described to possess insecticidal activity elicited by injection, and Mcf has also shown toxicity for NIH 3T3 cells (fibroblast cell line isolated from mouse). Txp also shows injection toxicity [17] and its suitability for development is yet to be established. 

From our data, we were not able to describe a common pattern of insecticidal genes distributed across species (Table 3). We hypothesize that the particular distribution of putative insecticidal genes and other virulence factors might depend on the presence of, and structural differences between, different megaplasmids harboured by the strain, as previously described in species belonging to the *Xenorhabdus* genus [44,45,46]. A similar diversity found over different insecticidal protein profiles is well known to be mediated and organized in megaplasmids in different insecticidal *B. thuringiensis* strains [47]. However, since our genomes are assembled into a draft state, the detection of novel plasmid sequences may be either inaccurately predicted or detected with poor confidence. To solve this problem, additional re-sequencing runs using long-read sequencing technologies such as PacBio or Oxford Nanopore will be necessary in order to close the circular chromosome and other extrachromosomal sequences. 

*X. szentirmaii* strains (Table 3) exhibit a variable number of genes encoding different toxin families; however, no strain of this species encodes Txp or Pra/Prb homolog proteins. In addition, there are between 5 and 17 Tc homologs and most strains encode one Mcf, whereas strain M encodes two and strain Cul lacks Mcf homologs, but is the only strain in this species to encode an App protein. Strains PSL and Reich, which belong to our novel proposed *X. littoralis* species, are inconsistent in the Tcs and Txp proteins encoded, whereas both encode Mcf homologs and show an absence of predicted Pra/Prb and App proteins. Strain 12, which belongs to our novel proposed species *X. santafensis* sp. nov., showed a low number of encoded insecticidal proteins, including just one copy of a Tc homolog, one Mcf and one App protein homolog, and it does not encode Txp or Pra/Prb proteins. Strain Vera, in contrast, encodes Tcs, Txp, Mcf and Pra/Prb homolog proteins but not App proteins. Strains 18, DI and 3, which have been classified as belonging to the species *X. doucetiae*, show a heterogenous set of encoded insecticidal proteins and an absence of encoded Txp and App proteins. Finally, the strain Flor belonging to the species *X. cabanillasi* was the only strain encoding the complete set of *Xenorhabdus* insecticidal proteins, including Tc proteins, Txp, Mcf, Pra/Prb and App proteins.

Along with the potential for encoding insecticidal proteins, the strains described here also encode putative chitinase enzymes and nematocidal proteins (Table 3). These enzymes produce the hydrolysation of the chitin, a major biological compound synthesised by crustaceans, insects and fungi [10]. Chitinases from *X. nematophila* have been proven to produce mortality in *H. armigera* when administered orally [10] and have also been described to enhance the activity of sub-lethal doses of Bt toxins by disrupting the insect’s peritrophic membrane [48]. In addition, chitinases from *X. szentirmaii* also showed a high capability to inhibit the growth of phytopathogenic fungi such as *Sclerotinia sclerotiorum* (Lib.) de Bary (Ascomycota: Sclerotiniaceae) [49]. 

Despite the fact that all of the strains studied in this work encode a different number of proteins exhibiting similarity with putative nematocidal protein 2 from *X. bovienii* (Acc. no. WP_012988617), it should be noted that no nematocidal activity has ever been reported for this protein, which would be better either renamed as putative nematocidal protein 2 or a hypothetical protein. 

As mentioned above, *Xenorhabdus* bacteria also produce bioactive secondary metabolites showing insecticidal and antimicrobial activities, with their synthesis pathways encoded for different gene clusters. Our genomic sequences encode a number of well-supported biosynthetic gene clusters showing similarity with those shown previously to produce known bioactive (antimicrobial) compounds. 

Potential applications in agriculture include the sustainable control of invertebrate pests, insect resistance management and the biological treatment of crop diseases (e.g., fungal and bacterial diseases). For example, *X. nematophila* is able to produce lysine-rich PAX peptides with strong antifungal activity against *Fusarium oxysporum* and other phytopathogenic fungi [50]. Other secondary metabolites such as fabclavines, rhabdopeptides and xenocoumacins have also been reported to show highly toxic activities against *Caenorabditis elegans* (Maupas) (Rhabditida: Rhabditidae) and *Meloidogyne javanica* (Treub) Chitwood (Tylenchida: Meloidogynidae) nematodes [51]. Bicornutin, fabclavines, rhabdopeptides and xenematides were also described to exhibit antimicrobial activities; however, rhabdopeptides and fabclavines have been also described for their cytotoxicity [52]. In a similar way to the difficulty in establishing a common pattern of distribution of insecticidal genes, we were unable to describe a clear correlation for the distribution of biosynthetic gene clusters, except for some duplications shared among some strains (Table 4). In addition, strain Vera showed a notable difference from the rest of the strains since it was the only one harbouring a secondary metabolite gene cluster for the biosynthesis of the Xenotetrapeptide compound. 

## 4. Conclusions

The biological control of pests and crop diseases is paramount in a transition towards environmentally friendly agriculture in order to suppress both crop pests and diseases that negatively affect crop production worldwide in a population that is steadily growing. In this study, we describe the genome sequence of fourteen *Xenorhabdus* strains and propose two novel species within the genus, providing a broader view of the diversity of the genus. We also identify a number of novel insecticidal gene sequences (and other virulence factors, including biosynthetic gene clusters) with the potential to be used for the biological control of invertebrate pests and crop diseases in agricultural and biotechnological applications.

## 5. Materials and Methods

### 5.1. Collection of Soil Samples and Isolation of Bacteria

To obtain representative soil samples, a total of 10 random sub-samples were collected from various locations in Argentina (Table 5). Each sub-sample was taken from a depth of 0–30 cm and combined to form a composite sample. Subsequently, the collected samples were introduced into 1-litre plastic pots, each containing 7th instar *G. mellonella* larvae. *G. mellonella* adults were collected from apiaries in the province of Santa Fe (Argentina) and reared at 28 °C in the dark; then, larvae were obtained from eggs up to the 7th instar, and were fed on a diet based on beeswax and pollen grain [53]. The pots were then inverted and placed in an incubator set at a temperature of 25 °C [54]. Dead larvae were identified after 7 days and individually placed in modified white traps [55]. Nematodes harbouring bacterial symbionts were first identified by morphological traits and placed in the intergeneric “glaseri group” [56]. Morphological and morphometric studies were later supplemented with molecular methods, including ITS and 28S rDNA gene sequence analysis, as previously described [57]. Each *Xenorhabdus* bacterial strain was then isolated from *G. mellonella* larvae cadavers using the following method: Dead larvae were surface-disinfected using 70% *v*/*v* ethanol and the insect haemolymph was extracted by puncturing the cuticle with a fresh (sterile) syringe needle. Then, a droplet of haemolymph was streaked onto Petri dishes containing NBTA agar (37 g nutrient agar, 25 mg bromothymol blue powder, 4 mL of 0.01 g/mL 2,3,5-triphenyltetrazolium chloride and 1000 mL distilled water) and incubated at a temperature of 28 °C for 48 h. Following the incubation period, those colonies exhibiting the characteristic morphological traits of *Xenorhabdus* were streaked onto a fresh NBTA plate to ensure axenic isolation [58]. The preliminary molecular characterization of each isolate was carried out by PCR amplification and the sequencing of the 16 S rDNA amplicon was carried out following the methodology described by Del Valle et al., 2016 [59]. 

### 5.2. DNA Isolation and Genome Sequencing

Each bacterial strain was grown in 5 mL Luria–Bertani (LB) broth (1% tryptone, 0.5% yeast extract and 1% NaCl, pH 7.0) with shaking at 200 rpm and 28 °C for 48 h in the dark, and then centrifuged at 5000× *g* for 5 min. Total genomic DNA was isolated and purified using the Wizard genomic DNA purification kit (Promega, Madrid, Spain), following the manufacturer’s instructions for Gram-negative bacteria. Genome sequencing was performed using high-throughput Illumina sequencing technology at the Wellcome Trust Centre for Human Genetics (London, UK). 

### 5.3. Genome Assembly and Annotation

The obtained (raw) Illumina reads were processed as follows: Reads were first trimmed and de novo assembled into contigs using the Velvet assembler 1.2.10 plug [60] included in Geneious R11 (www.geneious.com, accessed on 4 July 2023). Then, each whole-genome shotgun project was deposited at the DDBJ/EMBL/GenBank under the accession numbers described in the Data Availability Statement and automated annotations were provided by the NCBI Prokaryotic Genome Annotation Pipeline. The versions described in this paper are the first versions (e.g., JACXBB000000000.1). Multiple sequence alignments of putative pathogenicity islands showing gene arrangements and gene conservation were carried out with MAUVE [33]. 

### 5.4. Genome–Genome Comparisons and Phylogenetic Analysis

The phylogenetic relationships among the *Xenorhabdus* genomes were analysed with a modified multigene approach from Lee and Stock (2010) using the concatenated gene sequences of six housekeeping genes, including 16S rRNA, *recA*, *gyrB* (DNA gyrase subunit B), *dnaN* (DNA polymerase III), *gltX* (glutamate tRNA synthetase) and *infB* (translation initiation factor IF-2) [61]. The concatenated gene sequences (10,059 bp) were constructed using the Concatenate Sequences or Alignments tool included in Geneious R11. Multiple sequence alignments of each gene sequence and the resultant concatenations were obtained with the Muscle [62] plug included in Geneious R11. The best DNA model for multiple sequence alignments of concatenated sequences was searched with MegaX (version 10.2.6) [63]. The construction of the phylogenetic tree was performed using the maximum-likelihood method with a General Time Reversible model (GTR) plus the Gamma distribution and invariant sites (G+I) for estimating genetic distances. A total of 100 bootstrap replicates were performed for calculating branch quality. 

The genomes from different *Xenorhabdus* species were searched via the NCBI Taxonomy Browser and downloaded from the GenBank database [64]. Percentages of Genome Average Nucleotide Identity (% ANI) among genomes were calculated using the Enveomics ANI calculator tool (http://enve-omics.ce.gatech.edu/ani/index, accessed on 26 June 2023). The cut-off % ANI value for differentiation among genomes of different species is typically found to be below 95% ANI [65]. 

The type (strain) genome server (TYGS) [66] was used as a means of confirming the previous results shown by both the multigene phylogenetic approach and the % ANI value calculations.

### 5.5. Insecticidal Gene Annotation

Since specific insecticidal genes and other virulence factors are often missed or not accurately identified by the automated NCBI Prokaryotic Genome Annotation, additional searches were carried out using NCBI BLAST (Basic Local Alignment Search Tool) [67] with a custom insecticidal database and the RAST server [68]. 

The annotation of each putative insecticidal protein was then performed as follows: (i) Each predicted protein sequence was initially identified using BLASTP searches using a custom database including previously known insecticidal proteins from entomopathogenic bacteria [69]. (ii) Candidate protein sequences were later submitted to NCBI BLAST (blast.ncbi.nlm.nih.gov, accessed on 1 January 2024) with the Protein Data Bank database (PDB) selected [70]. (iii) Each identified insecticidal protein sequence was re-confirmed by searching the conserved domains in the Pfam database (http://pfam.xfam.org/, accessed on 4 July 2023) [71]. Finally, each putative insecticidal protein was also screened using the BestMatchFinder tool at the Bacterial Pesticidal Protein Resource Center (BPPRC) [30], and selecting to search by sequence option. 

### 5.6. Prediction of Biosynthetic Gene Clusters

A preliminary prediction of putative gene clusters involved in the production of bioactive secondary metabolites was automatically performed with the antiSMASH server [36]. Later, all gene clusters annotated by antiSMASH were manually curated by comparison with published gene clusters from well-studied *Xenorhabdus* strains [4,72] based on the Bode lab in-house database (Table 4) [35]. Identity or similarity to known BGCs was based on gene length, number of modules, or the modular architecture of the genes or BGCs.

## Figures and Tables

**Table 1:** 16S rRNA identification of isolated *Xenorhabdus* strains.

Strains (This Study)	Reference 16S rRNA	Nematode Host	GenBank Acc. No.	% Pairwise Identity
5	*X. szentirmaii*	*Steneinerma* sp.	FJ515803	99
Cul	*X. szentirmaii*	*S. rarum*	FJ515803	99
ZM	*X. szentirmaii*	*Steneinerma* sp.	FJ515803	99
M	*X. szentirmaii*	*S. rarum*	FJ515803	99
38	*X. szentirmaii*	*S. rarum*	FJ515803	99
42	*X. szentirmaii*	*S. rarum*	FJ515803	99
PSL	*X. szentirmaii*	*Steneinerma* sp.	FJ515803	98
Reich	*X. szentirmaii*	*Steneinerma* sp.	FJ515802	99
12	*X. mauleonii*	*Steneinerma* sp.	NR_043645	99
Vera	*X. szentirmaii*	*Steneinerma* sp.	FJ515802	99
18	*X. doucetiae*	*S. diaprepesi*	FO704550	99
DI	*X. doucetiae*	*S. diaprepesi*	DQ211702	99
3	*X. doucetiae*	*Steneinerma* sp.	DQ211702	99
Flor	*X. cabanillasii*	*Steneinerma* sp.	DQ211711	99

**Table 2:** Genome-based species identification using the multigene approach, % ANI calculations and TYGS server.

Strain	Multigene Approach	Species (%ANI)	Species TYGS	Proposed Species
*Xenorhabdus* sp. 5	*X. szentirmaii*	*X. szentirmaii* (99.47)	*X. szentirmaii*	*X. szentirmaii*
*Xenorhabdus* sp. Cul	*X. szentirmaii*	*X. szentirmaii* (99.65)	*X. szentirmaii*	*X. szentirmaii*
*Xenorhabdus* sp. ZM	*X. szentirmaii*	*X. szentirmaii* (99.64)	*X. szentirmaii*	*X. szentirmaii*
*Xenorhabdus* sp. M	*X. szentirmaii*	*X. szentirmaii* (99.64)	*X. szentirmaii*	*X. szentirmaii*
*Xenorhabdus* sp. 38	*X. szentirmaii*	*X. szentirmaii* (99.38)	*X. szentirmaii*	*X. szentirmaii*
*Xenorhabdus* sp. 42	*X. szentirmaii*	*X. szentirmaii* (99.74)	*X. szentirmaii*	*X. szentirmaii*
*Xenorhabdus* sp. PSL *	**Unknown**	**Unknown (≤85.00) ^a^**	**Unknown**	***X. littoralis***
*Xenorhabdus* sp. Reich *	**Unknown**	**Unknown (≤85.00) ^a^**	**Unknown**	***X. littoralis***
*Xenorhabdus* sp. 12	*X. mauleonii*	**Unknown (90.23, ≤85.00) ^b^**	**Unknown**	***X. santafensis***
*Xenorhabdus* sp. Vera	*X. koppenhoeferi*	*X. koppenhoeferi* (96.69)	*X. koppenhoeferi*	*X. koppenhoeferi*
*Xenorhabdus* sp. 18	*X. doucetiae*	*X. doucetiae* (97.25)	*X. doucetiae*	*X. doucetiae*
*Xenorhabdus* sp. DI	*X. doucetiae*	*X. doucetiae* (97.23)	*X. doucetiae*	*X. doucetiae*
*Xenorhabdus* sp. 3	*X. doucetiae*	*X. doucetiae* (97.25)	*X. doucetiae*	*X. doucetiae*
*Xenorhabdus* sp. Flor	*X. cabanillasi*	*X. cabanillasi* (97.46)	*X. cabanillasii*	*X. cabanillasi*

* PSL and Reich share 98.48% ANI with each other. ^a^ Strains showing ≤85.00% ANI against all the strains tested. ^b^ Strain showing 90.23% ANI against *X. mauleonii* and ≤85.00% ANI against the rest of the strains.

**Table 3:** General genome characteristics and insecticidal gene distribution of *Xenorhabdus* genomes used in this study, delimited by species.

Genomes														
Features	5	Cul	ZM	M	38	42	PSL	Reich	12	Vera	18	DI	3	Flor
Size (bp)	4,704,623	4,811,834	4,937,636	4,895,665	4,855,573	4,856,095	4,270,206	4,511,286	4,697,413	4,394,775	4,274,643	4,178,992	4,194,235	4,405,897
Contigs	439	809	675	594	396	684	629	336	550	701	674	466	513	553
CDs	4271	4246	4476	4485	4499	4329	3873	3899	4127	3861	3760	3756	3748	3908
GC%	43.7	43.3	43.7	43.6	43.9	43.6	43.1	43.6	43.4	43.0	45.0	45.4	45.4	42.8
Tc	8	9	7	5	17	8	-	8	1	2	7	6	6	2
Txp	-	-	-	-	-	-	1	-	-	1	-	-	-	1
Mcf	1	-	1	2	1	1	1	1	1	1	1	-	1	1
Pra/Prb	-	-	-	-	-	-	-	-	-	1	1	1	1	1
App	-	1	-	-	-	-	-	-	1	-	-	-	-	1
iProtein *^a^*	-	-	-	-	-	-	1	1	-	1	-	-	-	-
nProteins *^b^*	2	2	2	1	2	2	1	2	1	1	1	1	2	1
Chitinases	2	4	4	4	3	4	-	-	1	-	2	2	2	-
Species	*X. szentirmaii*						*X. littoralis*		*X. santafensis*	*X. koppenhoeferi*	*X. doucetiae*			*X. cabanillasi*

*^a^* iProtein: Unknown probable insecticidal protein annotated by RAST (contains a VRP1 super family and or a Neuraminidase Pfam conserved domain). *^b^* nProteins: Putative nematocidal proteins showing similarity to nematocidal protein 2 from *X. bovienii* (Acc. no. WP_012988617).

**Table 4:** Potential biosynthetic gene clusters and their distribution in each strain genome (grey boxes) detected with antiSMASH [34] by comparison against previously reported functional gene clusters. In cases where a gene cluster is found more than once in a genome, the quantity is numbered in the column.

	Strains														
Gene Clusters Manually Annotated from Bode’s Lab In-House Database [35]	Function	5	Cul	ZM	M	38	42	PSL	Reich	12	Vera	18	DI	3	Flor
Aryl polyene	Antioxidative														
β-lactone	Proteasome inhibitor														
GameXPeptide	Insect immunosuppressive														
Rhabdobranin	Unknown														
Xenorhabdin	Proteasome inhibitor														
PAX lipopeptides	Antifungal														
Lipocitide	Insect immunosuppressive														
Xenobactin	Antimicrobial														
Photoxenobactin	Siderophore/insecticidal														
Xenoamicin	Antiprotozoal														
Phenazine	Antibiotic		2			2	2								
Szentirazine	Unknown														
Fabclavine	Antimicrobial														
ATRed	Unknown		2		2	2	2	2	3	2					2
Pyrrolizixenamide	antibiotic														
Xenocoumacin	Antifungal/antibiotic														
Photopyrone	Quorum sensing														
Photobactin	Siderophore														
Safracin	Antibiotic														
Putrebactin/Avaroferrin	Iron uptake												2	2	
Xefoampeptide	Insect immunosuppressive														
Xenotetrapeptide	Insect immunosuppressive														
Species		*X. szentirmaii*						*X. littoralis*			*Xs*	*Xk*	*X. doucetiae*		*Xc*

Xs: X. santafensis, Xk: X. koppenhoeferi, Xc: X. cabanillasi.

**Table 5:** Geographical distribution of isolated strains and sample information.

Strain	City, Department, Province	Soil Sample
5	Videla, San Justo, Santa Fe	Soybean-cultivated soil
Cul	Cululú, Las Colonias, Santa Fe,	Corn-cultivated soil
ZM	Esperanza, Las Colonias, Santa Fe,	Corn-cultivated soil
M	La Criolla, San Justo, Santa Fe	Soybean-cultivated soil
38	Sarmiento, Las Colonias, Santa Fe	Corn-cultivated soil
42	Esperanza, Las Colonias, Santa Fe	Wheat-cultivated soil
PSL	Paso de los Libres, Paso de los Libres, Corrientes	Grassland soil
Reich	Gualeguay, Gualeguay, Entre Ríos	Soybean-cultivated soil
12	Marcelino Escalada, San Justo, Santa Fe	Native soil
Vera	Vera, Vera, Santa Fe	Native carob forest with cattle.
18	Colonia Campo del Medio, Garay, Santa Fe	Zucchini-cultivated soil
DI	Santa Rosa de Calchines, Garay, Santa Fe	Carrot-cultivated soil
3	Cabal, La Capital, Santa Fe	Soybean-cultivated soil
Flor	Florencia, General Obligado, Santa Fe	Sugar cane-cultivated soil

## Data Availability

The data described in this study can be accessed from the NCBI database via the following accession numbers: JACXBB000000000, JACXBC000000000, VCDO00000000, VCDP00000000, JACXBD000000000, JACXBE000000000, JACXBF000000000, JACXBH000000000, JACXBI000000000, JACXBJ000000000, VCDN00000000, JACXBK000000000, JACXBL000000000, JACXBG000000000.
